# Long Chain n-3 Fatty Acids Improve Depression Syndrome in Type 2 Diabetes Mellitus

**Published:** 2018-04

**Authors:** Maryam MAZAHERIOUN, Ahmad SAEDISOMEOLIA, Mohammad Hassan JAVANBAKHT, Fariba KOOHDANI, Mahnaz ZAREI, Samaneh ANSARI, Fatemeh KHOSHKHOO BAZARGANI, Mahmoud DJALALI

**Affiliations:** 1. Dept. of Cellular and Molecular Nutrition, School of Nutritional Sciences and Dietetics, International Campus, Tehran University of Medical Sciences, Tehran, Iran; 2. Dept. of Cellular and Molecular Nutrition, School of Nutritional Sciences and Dietetics, Tehran University of Medical Sciences, Tehran, Iran; 3. Dept. of Pharmacy, School of Medicine, Western Sydney University, NSW, Australia; 4. Endocrinology and Metabolism Research Center (EMRC), Tehran University of Medical Sciences, Tehran, Iran

**Keywords:** Depression, n-3 fatty acids, Type 2 diabetes mellitus, Randomized controlled trial

## Abstract

**Background:**

Type 2 diabetes mellitus (T2DM) is commonly associated with depressive symptoms, which affect prognosis and quality of life. We investigated the antidepressant effects of n-3 fatty acids (n-3FAs) monotherapy (without conventional antidepressants) for T2DM patients with mild to moderate depressive symptoms.

**Methods:**

A 10-wk, placebo-controlled, double-blind, parallel-group (1:1 ratio) randomized trial of n-3FAs (2700 mg/day EPA: DHA ratio=2) versus placebo in 88 Iranian diabetic patients with mild to moderate depression based on Beck Depression Inventory II (BDI-II-PERSIAN) was conducted. This study started from July 2014 to January 2015 in Tehran University of Medical Sciences, Tehran, Iran. The primary event was defined as worsened, non-changed, or inconsiderably improved depression (<5 unit decrease in BDI-II-PERSIAN depression scores after treatment) (ClinicalTrials.gov Identifier: NCT02261545).

**Results:**

Randomly, 44 T2DM patients were treated with n-3FAs supplements and 44 cases received placebo (three patients discontinued). n-3FAs could significantly protect patients against the aforesaid event and exhibit satisfactory prevention (number needed to treat with 95% confidence interval: 2.52, 1.71–4.74). No serious adverse reactions were reported.

**Conclusion:**

n-3FAs supplementation had significant antidepressant effects in T2DM patients with mild to moderate depressive symptoms, not confounded by metabolic factors and disease duration.

## Introduction

The paramount goal of “Standards of Medical Care in Diabetes” is to prevent or delay micro-and macro-vascular complications of type 2 diabetes mellitus (T2DM) through lowering glycated hemoglobin levels (HbA1C<7%) and tighter control of body weight, blood pressure, and circulating lipids (1). Unfortunately, few patients with T2DM can simultaneously achieve those recommendations (2). Comorbid depression in T2DM might be one of the possible causes of our failure to achieve sufficient glycemic control despite intensive medical treatment (3). Diabetes significantly increases the odds of having depression (3–5). Moreover, compelling evidence indicates that depression *per se* is one culprit for poor metabolic control and T2DM complications (3, 6, 7). This problem is elucidated as the interactive role of depression and diabetes in aggravation of T2DM because T2DM patients with depression do have diminished self-care and consequent poorer metabolic control, which results in increased diabetic complications and activation of a vicious cycle (6, 8, 9).

Conventional antidepressant treatment has been shown to ameliorate depression in T2DM patients, which may lead to improved glucose (10, 11). However, T2DM patients might not be able to easily tolerate conventional antidepressant agents; also these medications might exert disadvantageous metabolic effects (10, 11). Therefore, due to the harmful effects of depression on T2DM prognosis and diminished quality of life, it is necessary to develop effective anti-depression therapeutic strategies.

Long-chain polyunsaturated fatty acids (FA) of n-3 series (omega-3) including eicosapentaenoic acid (EPA) and docosahexaenoic acid (DHA, 22:6n–3) have been shown to play hypolipidemic and anti-inflammatory roles which lead to improvement of various metabolic characteristics (12). Potential antidepressant effects of n-3 fatty acids (n-3FAs) have been observed in patients with various types of depressive disorders (10, 13). n-3 and n-6 FAs are the essential components of phospholipids in cellular and subcellular membranes (14); which generate active metabolites and lipid mediators (e.g. prostaglandins, leukotrienes, protectins, endocannabinoids, N-acylethanolamines, etc.) playing autocrine and paracrine roles (14, 15).

There is a lack of evidence regarding the antidepressant effects of n-3FAs in T2DM patients. We aimed to evaluate the association between glycemic control and antidepressant effects of n-3FAs as the secondary goal.

## Materials and Methods

Overall, 88 patients with T2DM were recruited for this randomized, double-blind placebo-controlled clinical trial from the Iranian Diabetes Society during Jul 2014 to Jan 2015, based on the diagnostic criteria of fasting blood sugar (FBS) concentrations more than 126 mg/dl in two different times without using anti-glycemic medications. Inclusion criteria were willing to participate, men aged 30–65 yr old, and premenopausal women older than 30 yr, body mass index (BMI) from 25 to 40 kg/m^2^, avoidance of dietary supplements, vitamins and herbal products at least 3 months before and throughout the intervention, willing to maintain their current diet, and to sustain the current physical activity and lifestyle during the 10 wk of study, and having no special physical activities.

These patients were not included: patients consuming fish oil during the past 3 months, having chronic kidney disease, hepatobiliary diseases, GI diseases, hematological disorders, microalbuminuria, retinopathy, neuropathy, hypo- or hyper-thyroidism, type 1 diabetes (based on patient history and medical documentations), treatment with orlistat or sibutramine for weight loss, pregnancy and lactation, treatment with insulin or thiazolidinediones; using oral contraceptive medications, consuming dietary supplements, vitamins and herbal products 2 wk before study, and using non-steroidal anti-inflammatory drugs.

Participants were excluded when their anti-diabetic medications or the dosages were changed, in cases of new-onset inflammatory diseases requiring anti-inflammatory medications, if they were not willing to continue their participation in the trial, lack of adherence to intervention (based on weekly follow-up calls) defined as refusing to consume utmost 10% of recommended treatments, and in cases of any adverse reactions to medications.

The local Ethical Committee of Tehran University of Medical approved the study (Reference number 25367) and all patients provided written informed consent. This trial was registered ClinicalTrials.gov Identifier: NCT02261545. The specialists and staffs were blinded during trial; they called participants weekly to check their adherence to treatment.

### Interventions

The supplement group received n-3FAs (3 Soft gel/day recommended to consume with meals; each Soft gel contains 600 mg EPA and 300 mg DHA; Nutralab, Canada, Packaging in Zahravi Pharmaceutical. Co, Tabriz, Iran) for 10 wk, and the placebo group received 3 Soft gel/day (with meals; each Soft gel contains 1gr of edible paraffin; were provided by Minoo Pharmaceutical, Cosmetic and Hygienic Co., Iran. placebos were identical to supplement in terms of color, shape, and size) for 10 wk; both groups received their background therapies. Simultaneously, participants of both groups received their own anti-diabetic medications and were recommended to sustain their diets and physical activity.

### Randomization

Permuted block randomization sequences were created using Microsoft Excel version 2010 software. Sequentially numbered containers were generated for concealment by blinded statistician of project. The secretary of outpatient diabetes clinic also blinded, enrolled participants using sealed sequentially numbers with attached pockets containing medications (placebo and n-3FAs supplement were same in colour, smell, taste, and package), specialists of clinic blinded explained interventions to participants and followed their adherence by weekly calls and monthly visits. All adverse events were monitored and discussed by them.

### Outcomes and measurements

Event was defined as worsened, non-changed, or inconsiderably improved depression (<5 BDI-II-PERSIAN scores). BDI-II is a 21-item self-administered questionnaire that measures characteristic symptoms of depression. Score for each item ranges from 0 to 3, with a higher score indicating a greater problem. The total score ranges from 0 to 63. The validation and reliability of BDI-II were studied in Iranian population (16).

BDI-II-Persian has high internal consistency (Cronbach’s alpha=0.87) and acceptable test-retest reliability (r=0.74) (16). Absolute and relative risk reductions (ARR, RRR), and numbers need to treat (NNT) are employed for reporting the effects of n-3FAs intervention. Secondary outcomes include FBS, glycosylated hemoglobin (HbA1C), blood pressures, body mass index, waist and hip circumferences; and were collected according to standard protocols (17, 18). Physical activity of participants was assessed by validated Persian, last 7-day long form of International Physical Activity Questionnaire (IPAQ) (19).

### Statistical analyses

Quantitative data are described as mean ± SD. Within-group (before/after intervention) differences of were assessed by paired *t*-test. Between-groups differences were analyzed by Independent *t*-test, where indicated. Categorical data are reported as frequencies (percentages) and were tested either by χ^2^ or Fisher’s exact, where indicated. Binary logistic regression was employed to adjust for confounders regarding the association of treatments and event. *P-*values less than 0.05 were considered statistically significant, based-on two-sided tests.

## Results

### Participant flow

Two-hundred and three patients with T2DM were assessed for eligibility. One-hundred and fifteen patients were excluded for the following reasons: 77 of them did not meet the inclusion criteria, 28 ones refused to participate, and the remaining 10 patients for other reasons (e.g. traveling, become ill). Then, the remaining 88 T2DM patients (recruitment period: Jul 2014 to Jan 2015) were randomly (1:1) allocated to fish oil supplement or placebo and followed for 10 wk (until Apr 2015).

### Baseline Characteristics

Overall, patients were 62.4% male, 83.5% married, with a Mean ± SD age of 50.87 ± 7.30 yr. After randomized allocation, groups were not significantly different regarding demographics, anthropometric and laboratory measurements (Table 1). Eighty-five T2DM patients (44 in n-3FAs supplement group and 41 patients in placebo group) finished the trial and were analyzed.

**Table 1:** Baseline characteristics of patients with type 2 diabetes mellitus randomly allocated in Placebo and Treatment (n-3 fatty acids) interventions

Patients' Characteristics^*^		***Group***		***Difference#* P-*value***
		***Placebo***	***n-3FAs Supplement***	
Age (yr)		50.56 ± 7.2	51.15 ± 7.4	0.708
Sex	Male	24 (58.5)	29 (65.9)	0.483
	Female	17 (41.5)	15 (34.1)	
Body Mass Index (kg/m^2^)		29.21 ± 2.90	29.21 ± 3.58	0.991
Disease Duration (year)		7.57 ±2.2	6.93 ±1.82	0.147
Waist Circumference (cm)		102.49 ± 10.20	101.72 ± 10.57	0.734
Hip Circumference (cm)		106.09 ± 7.39	106.05 ± 7.92	0.985
Waist to Hip Ratio		0.96 ± 0.07	0.95 ± 0.05	0.564
FBS (mg/dl)		182.26 ± 52.48	172.11 ± 39.55	0.315
HbA1c (%)		7.84 ± 1.12	7.44± 1.08	0.101
SBP (mmHg)		130.72±12.53	128.47±10.35	0.367
DBP (mmHg)		84.79±79	83.63±63	0.569
Beck Depression Inventory score		17 ± 11	15 ± 9	0.431
Smoking	Never	31 (75.6)	39 (88.6)	0.074
	Occasional	4 (9.8)	0 (0.0)	
	Current smoker	6 (14.6)	5 (11.4)	
Activity	Low	29 (70.7)	22 (50.0)	0.115
	Medium	11 (26.8)	20 (45.5)	
	High	1 (2.4)	2 (4.5)	
Marital Status	Single	4 (9.8)	2 (4.5)	0.571
	Married	34 (82.9)	37 (84.1)	
	Widow	0 (0.0)	0 (0.0)	
	Separated	3 (7.3)	5 (11.4)	
Education	Illiterate	3 (7.3)	1 (2.3)	0.145
	Primary	7 (17.1)	10 (22.7)	
	Diploma	9 (22.0)	19 (43.2)	
	Bachelor	16 (39.0)	11 (25.0)	
	Master	6 (14.6)	3 (6.8)	
Job	Unemployed	15 (36.6)	17 (38.6)	0.845
	Employed	26 (63.4)	27 (61.4)	

* Quantitative variables are displayed as mean ± SD, and categorical variables are expressed by frequencies # Independent *t*-test was used for mean differences, chi-square and Fisher exact tests were used for categorical associations FBS, Fasting Blood Sugar ; HbA1C, Glycated Hemoglobin; SBP, Systolic Blood Pressure; DBP, Diastolic Blood Pressure

### Post-intervention Outcomes

**Fig. 1:**
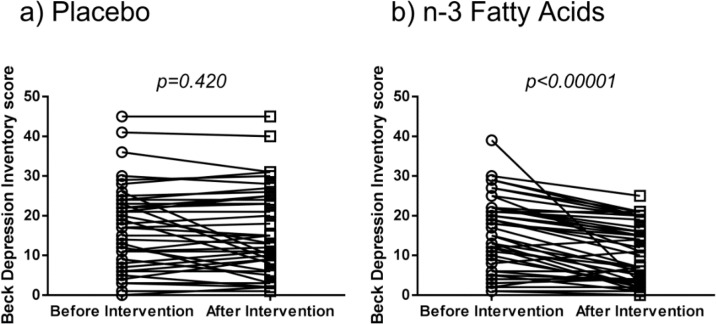
Changes in BDI-II scores of patients with type II diabetes mellitus before and after intervention

Fig. 1 shows changes of BDI-II scores of patients before and after interventions. Significantly, patients with T2DM treated with supplement showed decreased depression scores (34% improvement, *P*<0.001), while it was not significantly different before and after treatment for placebo. The information regarding the effectiveness of supplementation with n-3FAs for preventing the event is expressed in table 2. Satisfactory effectiveness was found as number needed to treat (NNT with 95% confidence interval) of 2.52 (1.71–4.74) (χ^2^=12.64; *P*=0.00038; OR with 95% confidence interval=0.156, 0.057–0.429) to prevent the event of worsened, non-changed, or inconsiderably improved depression (<5 unit decrease in BDI-II-PERSIAN depression scores after treatment).

**Table 2:** The effectiveness of treatments with n-3 fatty acids in prevention of the event defined as worsened, non-changed, or inconsiderably improved depression (<5 unit decrease in Beck Depression Inventory II scale) of type 2 diabetes mellitus patients

***Post-treatment Outcome***	***Placebo***	***n-3FAs Supplement***	***Total***
**Events**	34	19	53
Worsened, non-changed, or inconsiderably improved depression (<5 unit decrease in BDI-II-PERSIAN depression scores)			
**Non-events**	7	25	32
Considerably improved depression (≥5 unit decrease in BDI-II-PERSIAN depression scores)			
Total	41	44	85
Event Rate	82.93%	43.18%	
Absolute Risk Reduction (ARR) with 95% Confidence Intervals (95% CI)	39.75% (21.12%–58.37%)		
Relative Risk Reduction (RRR) with 95% CI	47.93% (24.89%–63.98%)		
Number Need to Treat (NNT) with 95% CI	2.516 (1.71–4.74)		
Relative Risk (RR) with 95% CI	0.521(0.348–0.780)		
Odds Ratio (OR) with 95% CI	0.156 (0.057–0.429)		
Chi-square *p*-value	0.00038 (χ^2^=12.64)		
BDI, Beck Depression Inventory			

Regarding side effects, one patient reported diarrhea self-limited, that was able to continue the trial.

To adjust for confounding effects, we used binary logistic regression (Table 3). After adjusting for the effects of sex, and glycemic control (defined by FBS<130 mg/dl and HbA1C<7%), the n-3FAs treatment remained significantly protective against the event of worsened, non-changed, or inconsiderably improved depression (*P*<0.001; OR with 95% confidence interval=0.086, 0.024–0.304).

**Table 3:** Adjustment for possible confounding variables by multivariate binary logistic regression regarding the effects of treatment with n-3 fatty acids on depressive symptoms of patients with type II diabetes mellitus

***Variables***	***Multivariate***				***Univariate***			
	**P-value**	**OR**	**95% CI for OR**		**P-value**	**OR**	**95% CI for OR**	
			**Lower**	**Upper**			**Lower**	**Upper**
Treatment	<0.001	0.086	0.024	0.304	<0.001	0.156	0.057	0.429
Sex (Female)	0.014	0.232	0.072	0.743	0.024	0.348	0.139	0.871
FBS <130 mg/dl	0.375	2.775	0.291	26.432	0.066	7.21	0.877	59.28
HbA1C <7%	0.103	2.936	0.805	10.706	0.179	1.99	0.728	5.473

FBS, Fasting Blood Sugar; HbA1C, Glycated Hemoglobin; OR, Odds Ratio; CI, Confidence Interval

Event is defined as worsened, non-changed, or inconsiderably improved depression (<5 unit decrease in BDI-II-PERSIAN depression scores after treatment)

## Discussion

This placebo-controlled double-blind randomized trial reveals that n-3FAs supplementation is effective to prevent development of depressive status of T2DM patients. If one hundred T2DM patients consume 2700 n-3FAs daily, 57 ones are prevented against the event (worsened, non-changed, or inconsiderably improved depression based on BDI-II-PERSIAN scores); 17 patients are prevented by themselves (regardless of n-3FAs), and 26 people are not protected. Interestingly, the protective role of n-3FAs supplements against depressive status was not confounded by glycemic control and other characteristics.

Compelling evidence supports the advantageous antidepressant effects of n-3FAs not only for major depression disorder (MDD) patients but also for mild depressive state (10, 13). It is of particular importance to note there are pathophysiological differences between MDD, depressive symptoms due to background (primary) disorders, and depressive patients without a diagnosis of MDD (13). It has been demonstrated for nondepressive healthy people, n-3FAs supplements have no beneficial effects regarding mood improvement (20–25). Nearly half of T2DM patients studied in our study were not depressed at baseline (based on the cut-off of 16 of BDI-II-PERSIAN scores (16) for Iranian population). Therefore, we were unable to consider depression/non-depression as the primary event of this study, then we defined it as worsened, non-changed, or inconsiderably improved depression scores (<5 unit decrease in BDI-II-PERSIAN depression scores after treatment). Moreover, severity of depressive status was assessed using averages of BDI-II-PERSIAN scores before/after treatment for this purpose; significantly decreased depression scores were observed. Although high doses of n-3FAs were not effective for improvement of depressive symptoms of healthy people and individuals with primary diseases (13), we found slight improvements in BDI-II scores of T2DM patients after 10 wk of supplementation with 2700 mg n-3FAs (EPA: DHA ratio of 2:1). n-3FAs supplements may alter depression and depressive symptoms in young adults with mild depression after a 21 d supplement (26).

The only RCT of add-on supplementation of EPA in diabetic patients with comorbid MDD showed no significant changes in Montgomery Åsberg Depression Rating Scale after 12 wk treatment with 1 g/day EPA, as compared to placebo (27). However, their study population was small (n=25) and heterogeneous with respect to the diabetes type and antidepressant treatment. They concluded the dose and duration of fish oil consumption might not have been sufficient (27). Add-on EPA supplementation in these patients showed limited effects on biological risk factors (28). EPA was only used as supplement, and they recommended using different concentrations of EPA in combination with DHA for future research. Our promising results might indicate that EPA: DHA ratio of 2:1 is more effective than EPA monotherapy.

The exact biological rational behind the antidepressant characteristics of n-3FAs remains unclear, but might be explained as the abundant presence and functions of polyunsaturated fatty acids (PUFA) in phospholipid structures of neuronal cell membranes in the brain, so that DHA and the n-6 fatty acid arachidonic acid constitute approximately 80% of total PUFAs in the brain (29). More interestingly, insufficient n-3FAs has been demonstrated to influence serotonin neuro-transmission in rats (30) so that synaptic levels of serotonin were changed both in basal conditions and after pharmacological stimulation with fenfluramine in rats with chronic dietary deficiency of n-3FAs. Moreover, concentrations of serotonin and dopamine metabolites in cerebrospinal fluid of healthy humans have been linked with DHA plasma levels (31). Briefly, the biochemistry of brain (i.e. arachidonic acid and DHA cascades, expression of cyclooxygenases and phospholipases A_2_, membrane structure, and membrane-related signaling pathways through cAMP, receptors, enzymes and etc.) are hypothesized to be influenced by n-3FAs supplements (10, 12, 32). One explanation for beneficial effects of n-3FAs might be their anti-inflammatory roles through the competition of DHA and EPA with arachidonic acid to be included in cell membrane, leading to lower production of eicosanoids and inflammatory cytokines derived from arachidonic acid (10, 12, 32). n-3FAs exert advantageous effects on metabolic characteristics (12, 17, 18, 33). Modulation of secretion of adipokines by dietary n-3FAs seems to play a key role for improvement of insulin sensitivity in muscle and liver through adiponectin-mediated AMP-kinase activation (12). Mild improvements in glycemic status and blood pressures of human individuals with metabolic syndrome and T2DM (12), might support the metabolic benefits of n-3FAs supplements in T2DM patients (17, 18). Further studies with long-term treatment periods are necessary for this purpose.

Strengths of our study are summarized as small loss to follow-up rate, homogeneous population with respect to diabetes type and medications, the double-blind randomized placebo-controlled design, sufficient sample size to detect mild changes, and using a reliable and valid instrument for depression measurement (BDI-II-PERSIAN). However, we faced some limitations, as we could not follow patients for a longer period. In addition, the majority of recruited patients had mild forms of depression. Unfortunately, we could not measure the n-3FAs levels in erythrocytes after treatment to verify compliance. In addition, our results are only generalizable to T2DM patients with mild to moderate depressive state receiving 2700 mg daily n-3FAs with EPA: DHA ratio of 2:1 with no comorbidities and no DSM-based diagnosis of MDD.

## Conclusion

This study indicated n-3FAs to be effective to significantly reduce depressive status of T2DM patients. The event of this study was defined as worsened, non-changed, or inconsiderably improved depression (<5-unit decrease in BDI-II-PERSIAN depression scores after treatment), significantly prevented in T2DM patients treated with n-3FAs supplements. These satisfactory antidepressant effects of n-3FAs were not confounded by other factors and did not induce adverse reactions. It seems early intervention with n-3FAs supplements is more effective to prevent MDD in T2DM individuals with mild to moderate depression, rather than treating comorbid MDD and T2DM. However, more long-term studies are necessary to confirm the effectiveness and safety of n-3FAs supplement in T2DM patients with depressive state.

## Ethical considerations

Ethical issues (Including plagiarism, informed consent, misconduct, data fabrication and/or falsification, double publication and/or submission, redundancy, etc.) have been completely observed by the authors.
